# Impaired immune surveillance exposes the germinal center as a niche for gammaherpesvirus-associated B cell lymphomagenesis

**DOI:** 10.3389/fimmu.2026.1859970

**Published:** 2026-10-16

**Authors:** Natalie Martinez, Brett A. Hoffman, Vasiliki Apostolou, April Feswick, Karl J. Ensberg, Mikayla S. Manzi, Vijay K. Sirohi, Yiping Wang, Karina E. Barbazuk, Mehmet Kara, Manu Nair, Rolf Renne, Erik K. Flemington, Genevieve M. Crane, Scott A. Tibbetts

**Affiliations:** 1 Department of Molecular Genetics and Microbiology, UF Health Cancer Institute, UF Genetics Institute, College of Medicine, University of Florida, Gainesville, FL, United States; 2 Department of Molecular Biology and Genetics, Bursa Uludag University, Faculty of Arts and Sciences, Bursa, Türkiye; 3 Department of Pathology, Tulane University School of Medicine, New Orleans, LA, United States; 4 Department of Pathology & Laboratory Medicine, University of California Irvine Medical Center, Irvine, CA, United States

**Keywords:** B cell, EBV, Epstein-Barr Virus, gammaherpesvirus, germinal center (GC) B cells, lymphoma, MHV68, murine γ-herpesvirus 68

## Abstract

**Background:**

Gammaherpesviruses establish lifelong latency in B cells and are linked to multiple human lymphoid malignancies, including Epstein-Barr virus (EBV)–associated lymphomas that arise at significantly increased frequency in settings of impaired immune surveillance. However, the cellular origins and evolutionary pathways of gammaherpesvirus-associated lymphomas remain incompletely defined.

**Methods:**

We used murine gammaherpesvirus 68 (MHV68) infection of β2m-deficient mice to reveal the pathological spectrum, differentiation states, and viral associations of gammaherpesvirus-driven malignancies arising under impaired immune control. Histopathologic and immunostaining analyses were used to characterize lymphoid neoplasms, B cell differentiation states, clonality, dissemination, and abundance of virus-positive cells.

**Results:**

MHV68 infection produced a broad spectrum of lymphoid neoplasms dominated by B cell lymphoproliferative diseases and B cell lymphomas. B cell lymphomas were the predominant lineage and exhibited morphologic and immunophenotypic features analogous to human EBV-associated diffuse large B cell lymphoma, follicular lymphoma, and marginal zone lymphoma. The majority displayed a germinal center B cell or post-germinal center phenotype, with 62% classified as aggressive rather than low-grade malignancies, and a subset showing clonal light chain restriction and systemic dissemination. MHV68-associated B cell lymphomas also exhibited marked heterogeneity in the abundance of virus-positive cells, including virus-rich and virus-poor tumors across multiple histologic subtypes. This pattern parallels the heterogeneity observed in EBV-associated malignancies and supports a model in which gammaherpesvirus-driven lymphomagenesis may proceed through both virus-dependent and virus-initiated pathways, including mechanisms resembling hit-and-run oncogenesis.

**Conclusions:**

These findings (a) further define the germinal center as a critical mutational niche in which gammaherpesvirus persistence and malignant transformation intersect, (b) reveal that gammaherpesvirus infection can give rise to diverse pathological outcomes even in genetically identical hosts, and (c) establish a framework for future translational studies of gammaherpesvirus-driven B cell lymphomas.

## Introduction

The seven known human tumor viruses are thought to directly contribute to greater than 13% of cancers worldwide (1). The human gammaherpesviruses Epstein–Barr virus (EBV) and Kaposi sarcoma–associated herpesvirus (KSHV) together account for a substantial fraction of these malignancies, including a diverse spectrum of lymphoid and epithelial tumors (2–4). EBV is associated with multiple B cell malignancies, including diffuse large B cell lymphoma (DLBCL), plasmablastic lymphoma, classic Hodgkin lymphoma, Burkitt lymphoma (BL), and lymphoproliferative disorders. EBV-associated malignancies (most notably endemic BL in sub-Saharan Africa, nasopharyngeal carcinoma in Southeast Asia, and gastric carcinoma) can develop in immunocompetent hosts. Nevertheless, many EBV-associated malignancies occur at increased frequency in settings of impaired immune surveillance, including post-transplant immunosuppression and HIV infection (2–5). KSHV is most strongly associated with Kaposi sarcoma, a tumor of endothelial origin, but also induces several lymphoid malignancies including primary effusion lymphoma (PEL), multicentric Castleman disease (MCD), and B cell lymphoproliferative disorders (2). Despite extensive molecular and cellular studies of these viruses, the *in vivo* mechanisms by which they establish lifelong infection and drive B cell lymphomagenesis remain poorly understood.

A central challenge in understanding gammaherpesvirus-associated malignancies is defining the cellular origins and evolutionary pathways of virus-associated tumors. Both EBV and the related murine gammaherpesvirus 68 (MHV68) establish lifelong latency within B cells that enter the germinal center program (6–8), a differentiation process characterized by rapid proliferation, somatic hypermutation, and clonal selection. Because germinal center B cells undergo extensive proliferation and mutation during affinity maturation, this critical process provides a context in which physiological disruptions can readily give rise to lymphomagenic outcomes (9). Accordingly, virus infections capable of expanding or prolonging germinal center reactions may create conditions that favor malignant clonal evolution, particularly when immune surveillance is impaired. Although extensive studies have defined molecular mechanisms governing gammaherpesvirus latency and host–virus interactions *in vitro*, the *in vivo* mechanisms by which gammaherpesviruses interact with germinal center processes to promote virus-driven malignancies remain poorly defined. Given the central role of the germinal center reaction in both gammaherpesvirus persistence and B cell lymphomagenesis, defining how virus-infected B cells behave within this specialized microenvironment *in vivo* is critical for understanding the origins of gammaherpesvirus malignancies.

Murine gammaherpesvirus 68 (MHV68) infection of mice provides a tractable experimental model for investigating these questions *in vivo* (10). MHV68 is a natural pathogen of rodents that is genetically and pathogenically related to EBV and KSHV (11, 12). Chronic MHV68 infection can induce B cell lymphoproliferative disease and lymphoma, particularly under conditions of immunosuppression such as cyclosporine treatment or genetic immunodeficiency (13, 14). Malignancies arising in these scenarios have been reported to histologically resemble human EBV-associated non-Hodgkin lymphoma and post-transplant lymphoproliferative disease (PTLD) (13, 14). However, the full pathological spectrum and differentiation state of MHV68-associated malignancies have not been systematically defined.

In work described here, we performed comprehensive histopathological and molecular characterization of MHV68-associated tumors arising in infected mice. Analysis of a large cohort of mock- and virus-infected animals revealed that MHV68 infection gives rise to a heterogeneous spectrum of lymphoid malignancies dominated by B cell lymphoproliferative disease and B cell lymphomas. Detailed phenotypic and molecular analyses demonstrated that the majority of B cell lymphomas exhibit a germinal center B cell phenotype and share morphologic and immunophenotypic features with several EBV-associated B cell lymphoma subtypes. Notably, these malignancies exhibit substantial heterogeneity in virus-positive cells, suggesting that gammaherpesvirus infection may contribute to B cell transformation and tumor evolution through both virus-dependent and virus-initiated pathways. Together, these findings provide *in vivo* evidence supporting the concept that germinal center biology represents a central nexus linking gammaherpesvirus latency with B cell lymphomagenesis, and reveal that gammaherpesvirus infection under conditions of impaired immune surveillance can give rise to diverse pathological outcomes.

## Materials and methods

Viruses. Three WT viruses were used to infect the cohort: MHV68 WT virus (15), MHV68.ORF73Bla, and MHV68.H2b.YFP. The recombinant marker viruses are phenotypically WT. MHV68.ORF73Bla expresses beta-lactamase fused to mLANA (16), MHV68.H2b.YFP expresses eYFP under the H2b promoter (17).

Mouse infections and organ harvests. Five- to six- week old C.129P2(B6)-B2mtm1Unc/J (β2m) mice were purchased from the Jackson Laboratory (Bar Harbor, ME) and housed in a University of Florida (UF) biosafety level 2+ facility. β2m mice lack CD8 T cells due to the requirement of β2m for the surface expression of classical MHC class I molecules during positive selection (18). Mice were infected intranasally (i.n.) with 10^5^ plaque-forming units (PFU) of virus in 20 μL serum-free DMEM under isoflurane anesthesia. Spleen, lung, and liver tissues from 198 MHV68-infected and 71 mock-infected mice were harvested at 0.3-18.3 months post-infection (mpi). Spleens were sectioned such that one-third was fixed in 10% neutral buffered formalin for histology and *in situ* hybridization. The remainder was flash-frozen in liquid nitrogen followed by -80 °C storage.

Histology and RNAscope *in situ* hybridization. Organs were embedded in paraffin, sectioned at 4 μm, and the resulting slides stored at 4 °C under desiccated conditions. Sections were either stained with hematoxylin and eosin (H&E) by the UF Molecular Pathology Core (Gainesville, FL) to assess general spleen architecture and metastasis, or deparaffinized for *in situ* hybridization for histopathological classification of splenic tumors. Hybridization was performed using Advanced Cell Diagnostics (ACD) RNAscope 2.5 HD Detection Reagents-RED (322360) and RNAscope 2.5 HD Duplex Detection Reagents (322500) according to manufacturer’s protocol. Pretreatment consisted of 15 minutes at > 95 °C for target retrieval and 30 minutes at  °C for protease treatment. MHV68-infected cells were detected using a custom RNA probe targeting MHV68 terminal repeats (446651 and 446651-C2). Initial tumor classification included the use of CD45/Ptprc (318651-C2) to identify leukocytes, CD3 (314721) for T cells, and CD19 (314711-C2) and PAX5 (311481) for B cells. B cell lymphoma subtypes were further classified using BCL6 (455311-C2) and MEF2B (1209121-C1) for germinal center (GC) B cells, CD138 (813921-C2), Ki67 (416771) for proliferation index, CD21 (549961-C2) for dendritic cell (DC) meshwork and DCs, CXCL13 (406311) for follicular dendritic cell meshwork and T follicular helper cells. IRF4 (494161-C2) and BCL2 (410751-C2) were used for further subclassification. CD10 was not used because it is not a reliable marker for GC B cells in mice. CD56 (497761) was used as a negative control (in mice it is a neural lineage marker but is not expressed in NK cells). Tumor clonality was inferred using immunoglobulin kappa (414291) and lambda (414301-C2) light chain probes. Virus-positive cells were identified using a probe to the MHV68 TR region. Images were acquired using a Nikon Eclipse E600 microscope with 4X, 10X, 20X, and 40X objectives and NIS-Elements imaging software (Nikon Instruments). An Olympus VS200 slide scanner was used for slide scanning. For images with significant yellow background post-acquisition, white balance correction was performed in Image J (19) using script from https://github.com/pmascalchi/ImageJ_Auto-white-balance-correction/tree/master. Correction did not alter image patterns or intensity.

To further refine lymphoma subclassification, B cell malignancies were blindly evaluated by a board-certified hematopathologist using integrated assessment of H&E morphology and RNAscope marker profiles. Samples were assessed in the context of diagnostic criteria used for human B cell lymphomas, and classification followed the recommended nomenclature by the 5th Edition WHO for LPD arising in the setting of immune deficiency and dysregulation (20).

RNA extraction and analysis. Flash frozen tissue fragments were ground up into a fine powder by mortar and pestle. Total RNA was extracted using TRIzol reagent (Invitrogen/Thermofisher) according to the manufacturer’s protocol. Pellets were reconstituted using RNase-free water, and total RNA was quantified. RNA purity and integrity was assessed using the Agilent bioanalyzer (Agilent Technologies). All laboratory procedures were carried out using water treated with 1% Diethyl pyrocarbonate (DEPC) (MP Biomedicals/Thermofisher), and surfaces were cleaned with RNaseZap (Ambion/Thermofisher). Prior to library preparation, RNA was DNase treated and rRNA was depleted using a NEBNext rRNA Depletion Kit v2 (New England Biolabs) to enrich both coding and noncoding transcripts. Libraries were prepped using the NEBNext Ultra II Directional Library Prep Kit for Illumina (New England Biolabs). Resultant libraries were sequenced using Dual-end, 150-bp sequencing on an Illumina Novaseq X instrument (Illumina). Reads were aligned and mapped to the mouse genome (GRCm38) using STAR aligner (21) with default settings. Aligned reads were quantified using featureCounts (22) to produce raw reads. To account for differences in sequencing depth and library composition, raw counts were then median-of-ratios normalized by DESeq2 (23). Resultant counts were then used for RNAseq based ABC/GCB classification as described by Reddy et al., 2017 (24). Briefly, reads were log2 transformed and z-normalized across previously identified ABC and GCB associated genes (25). Z-scores were averaged across ABC and GCB associated genes then subtracted to generate a combined subtype score (**Supplementary Table 1**), where samples scoring above 0.25 were classified as ABC and below -0.25 as GCB.

Statistical analyses. All data were analyzed using Prism 11 software (GraphPad, San Diego, CA).

Statistical significance was determined using Pearson’s chi-square test, Brown-Forsyth and Welch’s ANOVA with Dunnett’s T3 multiple comparison test, or two-tailed, unpaired Welch’s t test. P values less than 0.05 were considered to be statistically significant (ns = not significant, *P < 0.05, **P < 0.01, ***P < 0.001, and ****P < 0.0001).

Study approval. All procedures were approved by the UF Institutional Animal Care and Use Committee (IACUC) in accordance with federal guidelines.

## Results

### MHV68 infection produces a spectrum of lymphoid malignancies

To assess long-term pathogenesis following a physiologically relevant intranasal route of infection, 269 β2m-deficient mice were inoculated intranasally with WT MHV68 (n= 198) or mock inoculum (n= 71) and monitored for up to 18 months post-infection (mpi). Although the target harvest window was 8–10 mpi, some animals reached humane endpoints earlier, and others were retained longer to capture late-onset malignancies. At harvest, spleen (typical primary site of disease), lung, and liver tissues were formalin-fixed, paraffin-embedded, and sectioned for histopathology and RNAscope *in situ* hybridization (ISH). For some samples, portions of the spleen were dissected and flash frozen prior to fixation.

Hematoxylin and eosin (H&E) staining of spleen sections revealed three major pathological outcomes following infection: lymphoid hyperplasia (LH), lymphoproliferative disease (LPD), and lymphoma (**Figures 1A**; **Supplementary Figure 2**). LH was operationally defined by expansion of lymphoid follicles or white pulp, with preservation of overall splenic architecture and distinction between white and red pulp. LPD was defined by marked, coalescent lymphoid expansion, with partial or complete effacement of the splenic architecture, evidenced by diminished or absent distinction between white and red pulp, and progressive loss of red pulp. In contrast, lymphoma samples demonstrated complete architectural effacement. Further immunostaining analyses of a subset of LPD samples (not shown) demonstrated areas of skewed clonal proliferation, supporting the presence of an LPD over LH. Aggressive LPD or large cell lymphoma were identified by the presence of sheets of large cells. Classification followed the recommended nomenclature by the 5^th^ Edition WHO for LPD arising in the setting of immune deficiency and dysregulation (20).

**Figure 1:**
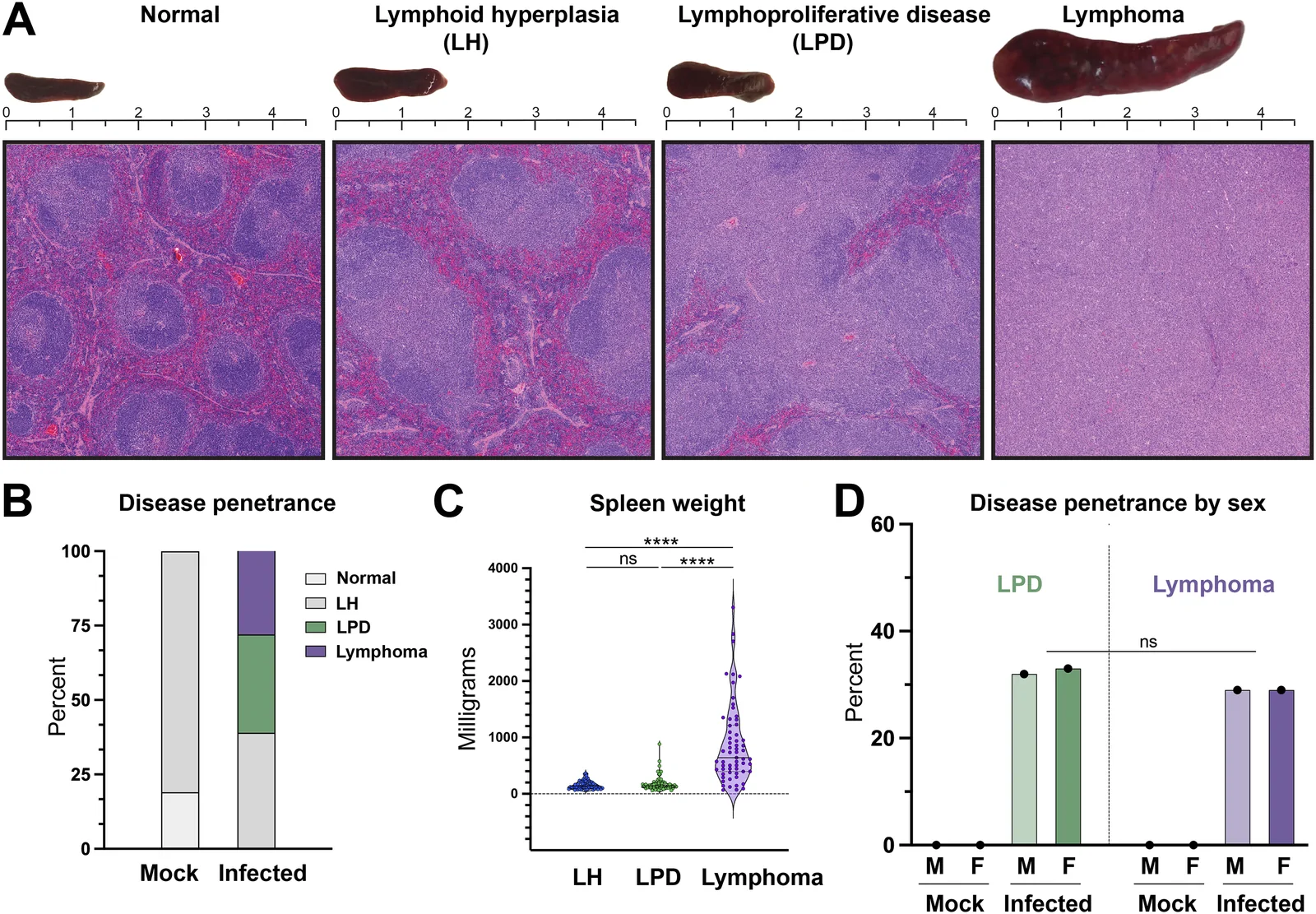
Penetrance and general features of MHV68-associated lymphoproliferative diseases. **(A)** Representative gross images of spleens and hematoxylin and eosin (H&E)-stained spleen sections showing major histopathological outcomes following MHV68 infection: lymphoid hyperplasia (LH), lymphoproliferative disease (LPD), and lymphoma. A normal spleen from a mock-infected animal is shown as a control. All images are shown at 4x magnification. **(B)** Disease penetrance 8-10 months post-infection (mpi) in mock- and MHV68-infected mice (mock n= 44, infected n= 80). The distribution of disease was dependent on infection status (χ^2^(2, N=124) =44.56, p = <0.0001). **(C)** Spleen weights from MHV68-infected mice stratified by disease state. Center solid line represents the median, each dot represents an individual mouse. Significance determined by Brown-Forsythe and Welch's ANOVA with Dunnett's T3 multiple comparison test (ns, not significant, ****P<0.0001). **(D)** Disease penetrance from mice in panel **(B)** stratified by sex (M, male; F, female). The distribution of disease was not dependent on sex (χ^2^(1, N=49) =0.01370, p = 0.9068).

At 8–10 mpi, MHV68 infection resulted in high penetrance of lymphoproliferative disease (**Figure 1B**). Mock-infected animals (n= 44) exhibited normal or LH outcomes (normal 21%; LH 79%), with no LPD or lymphomas observed. In contrast, among 80 virus-infected mice, LPD and lymphoma occurred at frequencies of 33% and 29%, respectively, with the remaining exhibiting LH. An additional 11 spleens (10 infected, 1 mock) displayed atypical extramedullary hematopoiesis (EMH) and partially disrupted architecture that did not meet criteria for LPD or lymphoma, and thus were excluded from the study. Spleen weight correlated with disease severity (**Figure 1C**), with lymphoma samples exhibiting significant variability and including several cases of pronounced splenomegaly (mean: 880.5 mg) as compared to LPD (mean: 185.3 mg), LH (mean: 157.7 mg) and normal (mean 126.8; not shown) samples. No significant sex-specific differences were detected (**Figure 1D**). These findings demonstrate that MHV68 infection in β2m-deficient mice induces lymphoproliferative disorders and lymphoma at high frequency.

### The majority of MHV68-associated malignancies are B cell lymphomas that demonstrate a germinal center phenotype

To determine the cellular origin of these malignancies, RNAscope *in situ* hybridization (ISH) for lineage markers was performed on 58 of the 61 malignancies identified by gross histopathological examination (from full cohort of 198 infected mice). ISH lineage marker probes used in the initial analysis included CD45 (leukocytes), CD19 (B cells), and CD3 (T cells). CXCL13 staining for follicular dendritic cell meshwork was used to visualize follicular architecture. For these experiments, RNAscope ISH was utilized to permit multiplex compatibility of cell lineage markers with virus genome detection, as this method allows consistent detection of virus-positive cells even in highly restricted and variable states of latent virus gene expression.

Among all malignancies, 95% (n = 55) exhibited moderate to high expression of the leukocyte marker CD45 and were therefore classified as lymphoma. Of these, B cell lymphomas represented the predominant malignancy. As compared to LH controls, B cell lymphomas exhibited a range of histopathologic severity, with advanced lesions showing marked expansion of CD19^+^ B cells, loss of CXCL13-defined follicular structures, and complete effacement of splenic architecture (**Figure 2A**). A smaller number of samples that were initially scored as malignancies exhibited prominent CD3 staining consistent with T cell lineage or were not classifiable based on the initial marker panel; these samples were not examined in further detail. Overall, B cell lymphomas accounted for 65.5% of total tumors (**Figure 2B**). These findings indicate that MHV68 infection produces a spectrum of hematologic malignancies in which B cell lymphomas represent the dominant tumor type.

**Figure 2:**
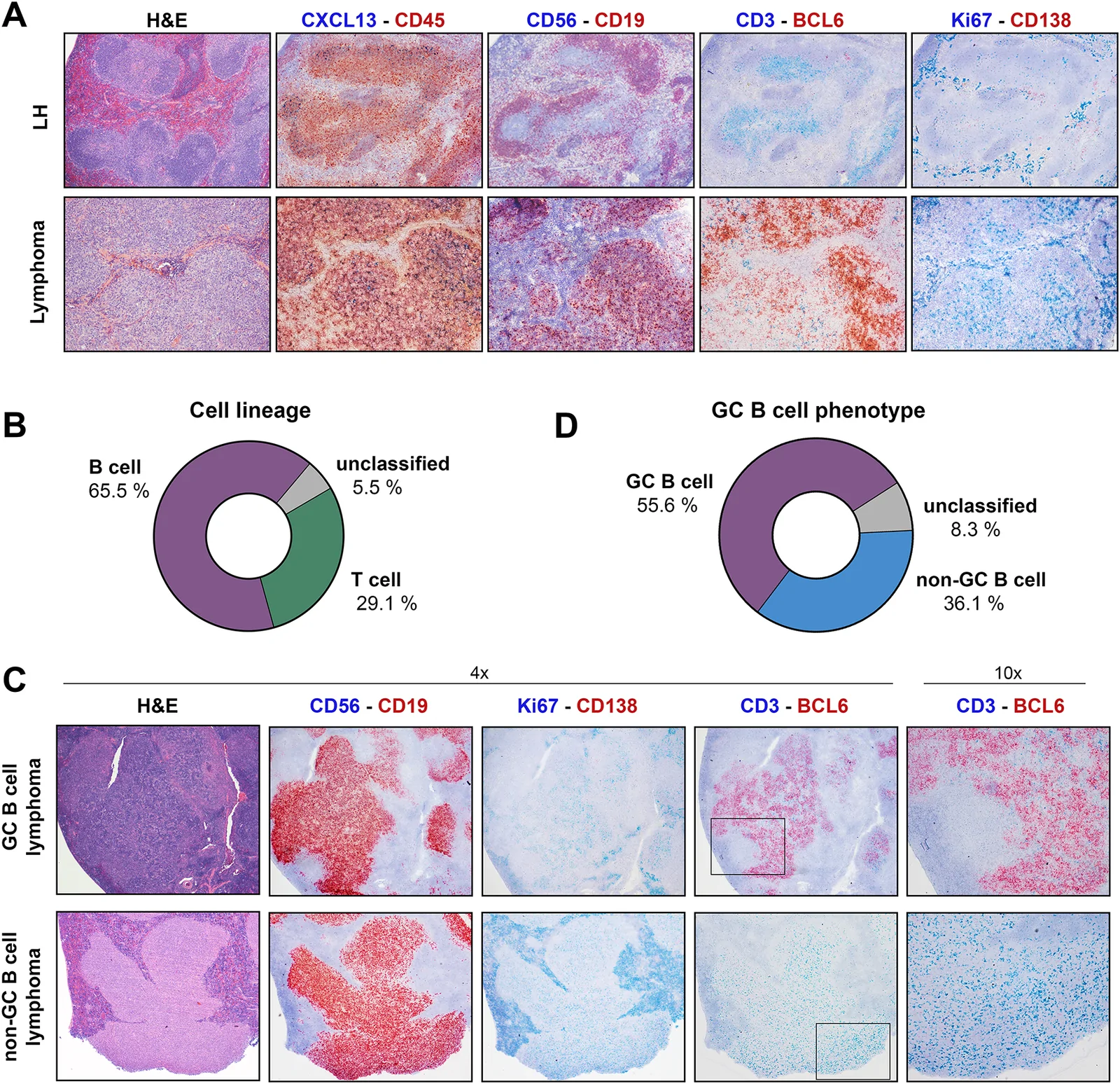
Most MHV68-associated malignancies are B cell lymphomas that exhibit a germinal center B cell phenotype. **(A)** Representative images of LPD and lymphoma. H&E staining shows overall tissue architecture. RNAscope ISH staining reveals follicular dendritic cell meshwork (CXCL13), leukocytes (CD45), B cells (CD19), germinal center B cells (BCL6), T cells (CD3), plasma cells (CD138), and proliferation index (Ki67). CD56 was used as a negative control probe. All images are shown at 4x magnification. **(B)** Quantification of cell lineage of MHV68-induced malignancies as determined by cell lineage marker staining (n= 55 total, 36 B cell, 16 T cell, 3 unclassified). **(C)** Representative images of germinal center (GC) and non-germinal center (non-GC) B cell lymphomas. All images are shown at 4x magnification unless otherwise indicated. Rectangles indicate the locations of 10x images. **(D)** Quantification of B cell malignancies exhibiting germinal center phenotype (n= 36 total, 20 GC, 13 non-GC, 3 unclassified).

To further define the differentiation state of B cell malignancies, parallel spleen sections were analyzed using RNAscope panels to assess lineage markers (CD45, CD19, PAX5, CD3), germinal center markers (BCL6), plasma cell markers (CD138), follicular architecture (CXCL13, CD21), and proliferation index (Ki67). Among B cell lymphomas, a majority expressed moderate to high levels of BCL6 (**Figure 2C**, top row), consistent with a germinal center B cell phenotype. Germinal center B cell phenotype was further validated by staining for MEF2B, a transcription factor critical for germinal center B cells, and by co-staining for BCL6 and CD3 to rule out BCL6+ T cells (**Supplementary Figure 2**). CD10 was not used for these studies because it is not a reliable germinal center B cell marker in mice. Within tumor samples, BCL6 expression was frequently associated with follicular structures, although in some tumors it displayed an extrafollicular distribution across the tissue. A smaller fraction of tumors exhibited absent or diminished BCL6 expression (eg, **Figure 2C**, bottom row), suggesting a non–germinal center B cell origin. Overall, 55.6% of tumors exhibited a germinal center B cell phenotype (**Figure 2D**), indicating a germinal center origin for the majority of MHV68-associated B cell lymphomas.

### MHV68-associated malignancies exhibit heterogeneous viral genome abundance

To define the distribution of virus-infected cells within malignancies, RNAscope ISH targeting the MHV68 terminal repeat (TR) region was performed to detect viral genomes. The TRs were used for this method because they are readily detectable repeat sequences that are present even during the restricted gene expression programs operational during latency. Co-staining with CXCL13 enabled visualization of follicular dendritic cell networks and germinal center architecture (**Figure 3A**). In LH samples, TR-positive cells were primarily localized within germinal centers, typically appearing as discrete clusters within individual follicles. In contrast, LPD and lymphoma samples exhibited highly variable viral staining patterns, ranging from tumors with abundant virus-positive cells distributed throughout the tissue to tumors containing only rare virus-positive cells.

**Figure 3:**
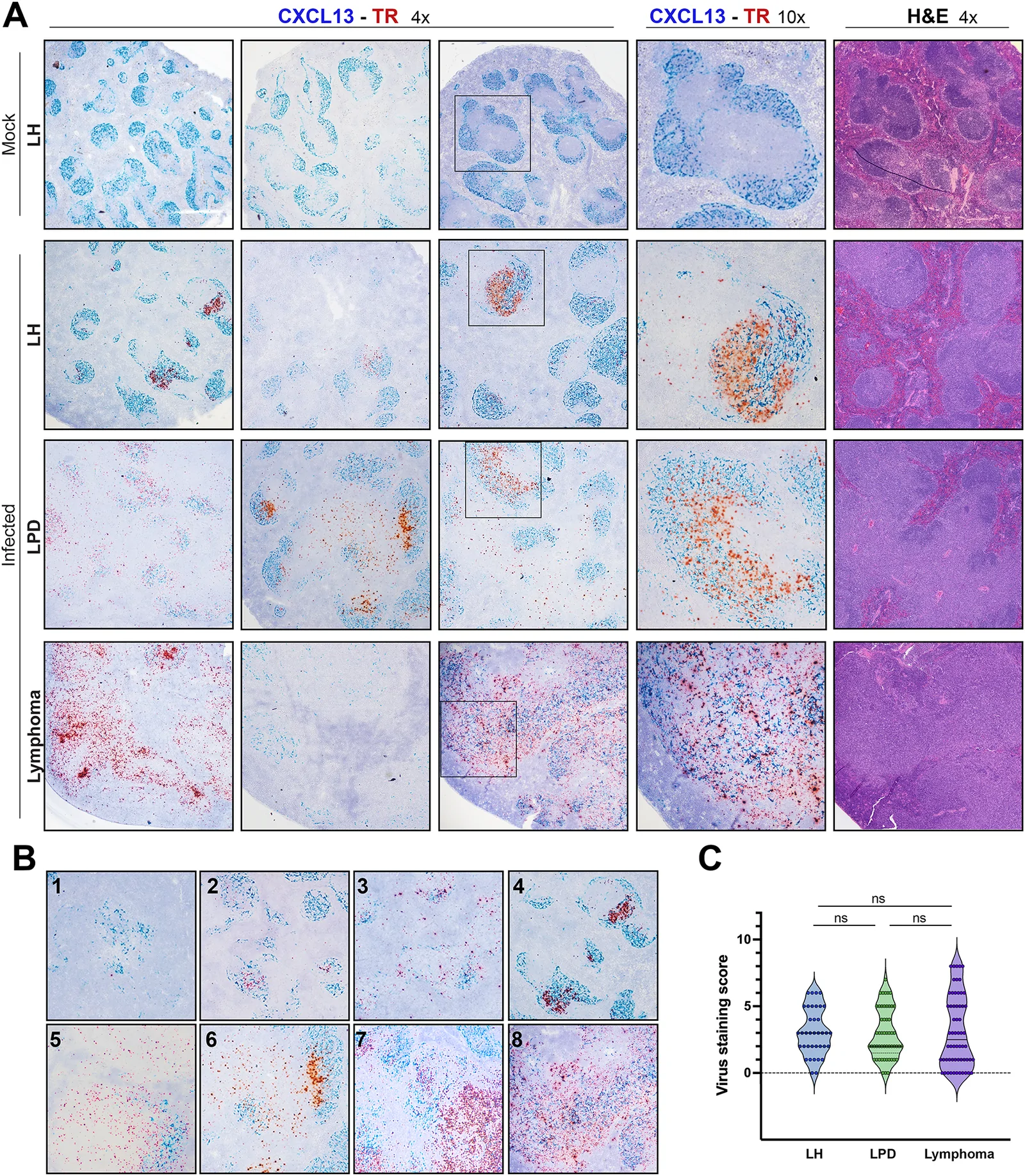
B cell malignancies exhibit heterogeneous abundance and distribution of virus-positive cells. RNAscope in situ hybridization using probes directed against the MHV68 terminal repeat (TR) region was used to detect MHV68-positive cells in tissue sections. **(A)** Representative images illustrating virus staining patterns across disease categories, including LH, LPD, and lymphoma. In LH samples, virus-positive cells predominantly localized to germinal centers. In LPD and lymphoma samples, viral staining was highly variable, ranging from rare puncta to widespread or clustered staining. **(B)** Scoring rubric used to quantify viral staining. Representative images are shown for each virus staining score (1 to 8), based on number and distribution of TR-positive cells (see text for details). Negative samples were scored 0. **(C)** Virus staining scores stratified by disease category. Each dot represents and individual sample. Center line indicates median. Significance determined by Brown-Forsythe and Welch's ANOVA with Dunnett's T3 multiple comparison test (ns, not significant).

To facilitate comparison across samples within the cohort, a semi-quantitative blind scoring rubric incorporating both TR staining abundance and spatial distribution was applied to all lymphoma and control samples (**Figure 3B**). Thus, TR staining in samples ranged from low and dispersed TR+ puncta (score 1-2), to TR+ small clusters (score 3), diffuse TR+ staining across the tissue (score 4), increased but inconsistent TR+ clusters (score 5), numerous TR+ clusters (score 6), strong TR+ clusters with consistent TR+ puncta across tissue (score 7), consistent very strong TR+ puncta and/or clusters across the entire tissue (score 8). Virus staining scores were stratified by disease category (**Figure 3C**); however, no significant difference was detected among total LH, LPD and lymphoma cohorts. Notably, B cell lymphomas displayed a wide range of virus staining, suggesting underlying biological heterogeneity among these tumors.

### MHV68-associated B cell lymphomas exhibit clonal expansion and metastatic dissemination

The unique B cell receptor present on each cell is comprised of a rearranged immunoglobulin heavy chain carrying a unique variable region, which is associated with an immunoglobulin kappa light chain (Igκ) or lambda light chain (Igλ). In human tumor histopathology, the Igκ:Igλ ratio is frequently used as a means to infer the clonality of B cell tumors when the ratio significantly deviates from normal values of 1.5:1 to 2:1 (26, 27). Although in the B cell population of healthy adult mice Igκ predominates with approximately 95% utilizing kappa light chains and ~5% utilizing lambda light chains (28), clonality of murine B cell tumors can still be inferred in scenarios in which Igλ is uniform across malignant cells. Thus, clonality of MHV68-associated B cell tumors was assessed using RNAscope probes targeting Igκ and Igλ.

Consistent with the light chain distribution in normal mice, LH samples (**Figure 4A**) displayed robust Igκ staining, primarily within B cell follicles, but also interspersed within extrafollicular regions, with only rare Igλ-positive cells detectable. LPD samples (**Figure 4B**) showed greater variability in distribution but typically exhibited widespread Igκ staining throughout atypical hyperplastic regions, with some cases demonstrating increased numbers of extrafollicular Igκ-positive cells. Occasional LPD samples (eg, **Figure 4B**, right side) contained increased but still relatively low numbers of Igλ-positive cells. A fraction of B cell lymphomas exhibited similar Igκ restriction (**Figure 4C**, left side). However, other B cell lymphomas exhibited pronounced expansion of Igλ -positive cells alongside Igκ cells (**Figure 4C**, right side) or strong Igλ restriction with few Igκ-positive cells (**Figure 4D**), consistent with monotypic expansion of an Igλ-restricted malignant B cell clone.

**Figure 4:**
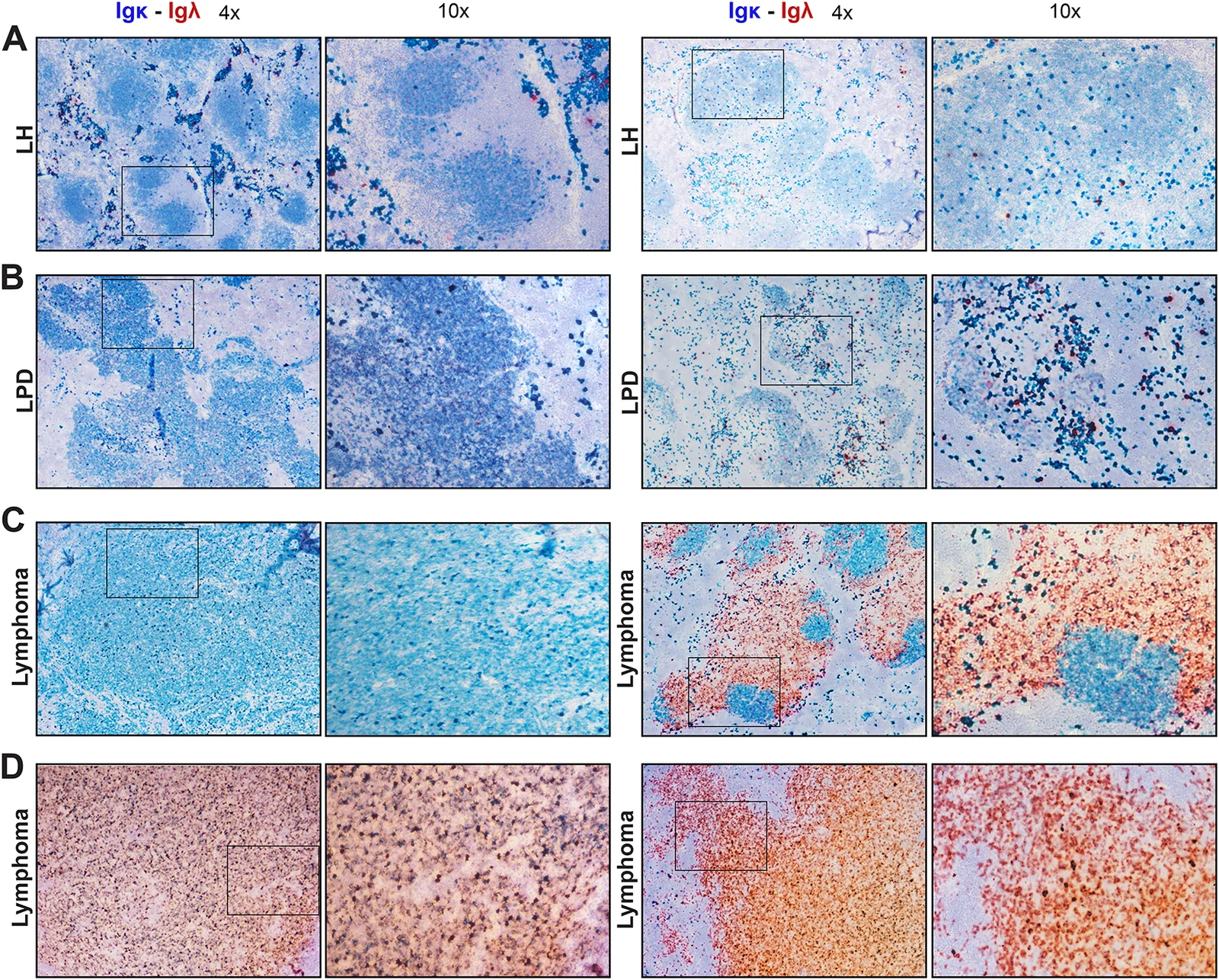
A subset of MHV68-associated B cell lymphomas exhibit clonal expansion of Igλ-positive B cell populations. Representative images from **(A)** 2 LH samples, **(B)** 2 LPD samples and **(C, D)** 4 lymphoma samples showing RNAscope in situ hybridization staining for immunoglobulin light chain kappa (Igκ; blue) and lambda (Igλ; red). For each sample, left panels are shown at 4x magnification, right panels at 10x magnification. In mice, the normal Igκ/Igλ ratio is approximately 95/5. All LH and LPD samples exhibited prominent Igκ staining [eg, panels **(A, B)**]. Some lymphomas exhibited prominent Igκ staining [eg, panel **(C)**, left side]. In contrast, a subset of B cell lymphomas exhibited a pronounced expansion of Igλ-positive cells alongside Igκ cells [eg, panel **(C)**, right side], or overall high frequencies of Igλ-positive cells with few Igκ-positive cells [eg, panel **(D)**], consistent with expansion of an Igλ-positive B cell clone.

In addition to clonality, advanced B cell lymphomas commonly demonstrated metastatic involvement of lung and liver (**Figure 5**), characterized by marked infiltration of CD19^+^ B cells (frequently Igλ-restricted) as compared to LH controls. Together, these findings infer that MHV68-associated B cell lymphomas represent clonally expanded malignancies capable of systemic dissemination.

**Figure 5:**
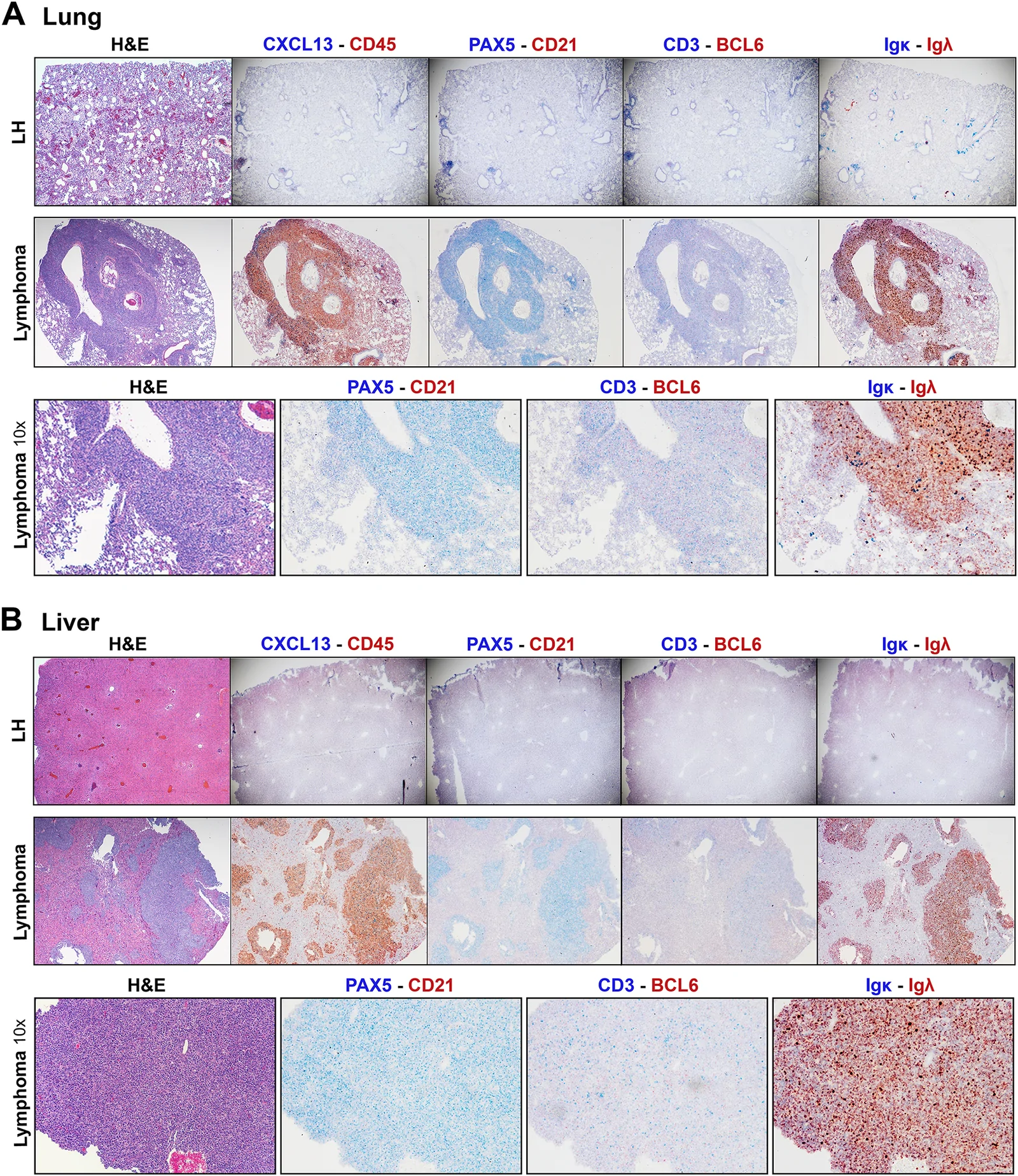
Advanced B cell lymphomas exhibit systemic dissemination to lung and liver. Representative **(A)** lung and **(B)** liver sections from mice with splenic LH or advanced splenic B cell lymphoma. RNAscope in situ hybridization staining reveals follicular dendritic cell meshwork (CXCL13), leukocytes (CD45), B cells (PAX5), T cells (CD3), germinal center B cells (BCL6), and B cells positive for immunoglobulin light chain kappa (Igκ) and lambda (Igλ). All images are shown at 4x magnification unless otherwise stated.

### MHV68-induced B cell lymphomas resemble human EBV-associated lymphoma subtypes

To further refine lymphoma subclassification, a subset of 29 B cell malignancies from the full cohort was blindly evaluated by a board-certified hematopathologist using integrated assessment of H&E morphology and RNAscope marker profiles. Samples were assessed in the context of diagnostic criteria used for human B cell lymphomas, and classification followed the recommended nomenclature by the 5th Edition WHO for LPD arising in the setting of immune deficiency and dysregulation (20). MHV68-associated B cell lymphomas predominately featured morphological and immunophenotypic features consistent with marginal zone-like lymphomas (MZL), follicular lymphomas (FL), and diffuse large B cell lymphomas (DLBCL).

Marginal zone-like lymphomas were typically characterized by retained nodular white pulp architecture, perifollicular expansion of B cells (frequently clonally restricted), low BCL6 expression, frequent plasmacytic differentiation, and a marginal zone–like growth pattern (**Figure 6**). In some MZL samples, focal areas with large transformed cells were apparent, raising the possibility of simultaneous transformation toward DLBCL. Notably, while strong virus-positive staining was observed in several MZL (eg, **Figure 6A**), virus-negative MZL were also observed (eg, **Figure 6B**), indicating that MZL can occur across a range of viral burdens.

**Figure 6:**
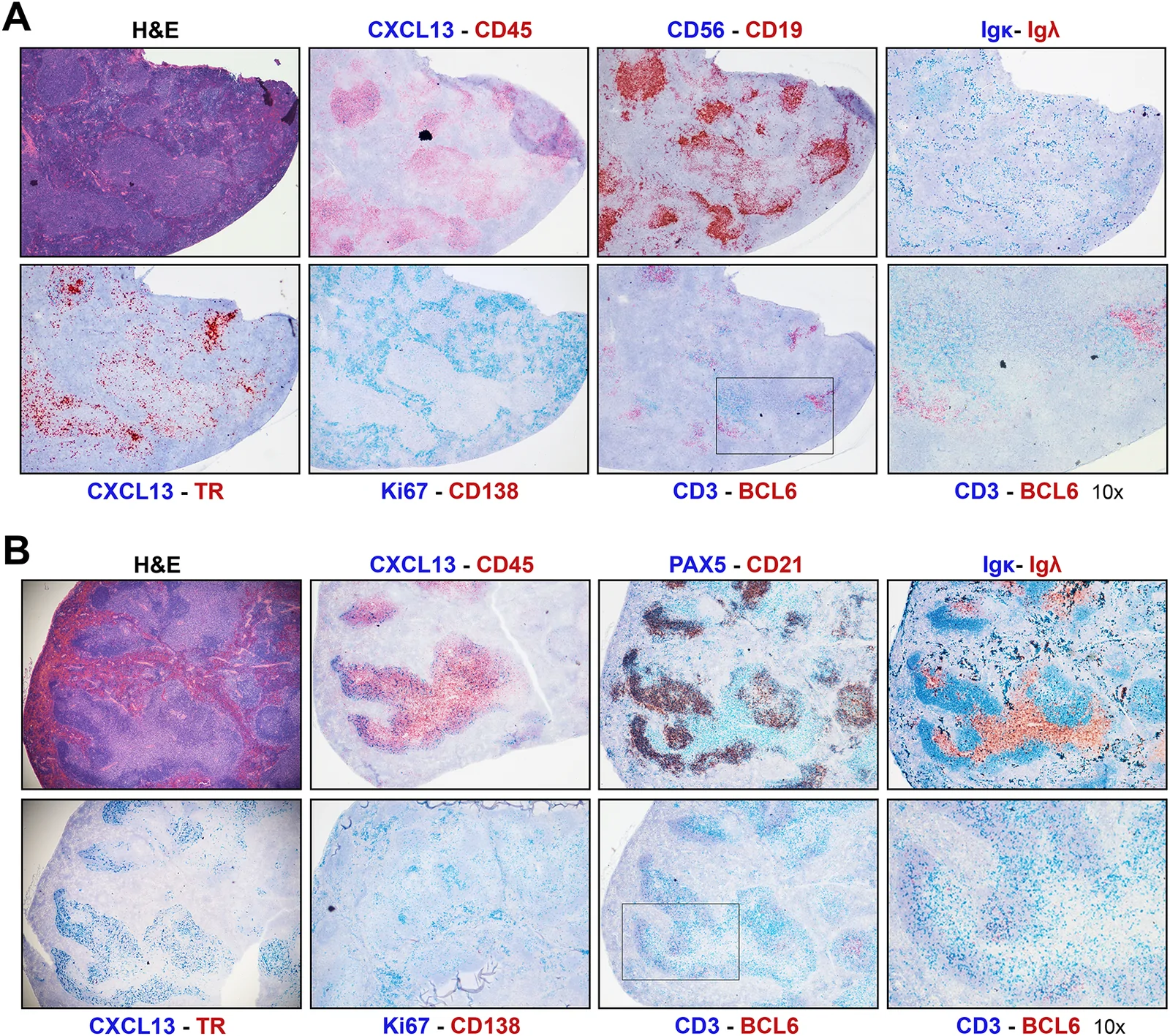
MHV68 induces virus-positive and virus-negative marginal zone-like B cell lymphomas. Representative images of **(A)** virus-positive and **(B)** virus-negative marginal zone-like B cell lymphomas. RNAscope in situ hybridization staining reveals follicular dendritic cell meshwork (CXCL13, CD21), leukocytes (CD45), B cells (CD19, PAX5), B cells positive for immunoglobulin light chain kappa (Igκ) or lambda (Igλ), plasma cells (CD138), T cells (CD3), germinal center B cells (BCL6), proliferation index (Ki67), and virus-positive cells (TR). CD56 was used as a negative control probe. All images are shown at 4x magnification unless otherwise stated. Rectangles indicate the locations of 10x images.

Follicular lymphomas (**Figure 7**) exhibited morphologic and immunophenotypic features consistent with FL, including follicular growth patterns and elevated proliferation indices. For example, the sample depicted in **Figure 7A** exhibited effaced architecture with back-to-back nodular lymphoid proliferation composed predominantly of large, atypical BCL6-positive cells with prominent nucleoli, co-localized virus signal, and high proliferative activity, favoring a virus-positive FL-like lesion with grade 3B features. An additional sample (**Figure 7B**) featured large atypical follicles with scattered CXCL13-positive meshworks and BCL2-supported follicular differentiation, consistent with FL despite weaker BCL6 staining.

**Figure 7:**
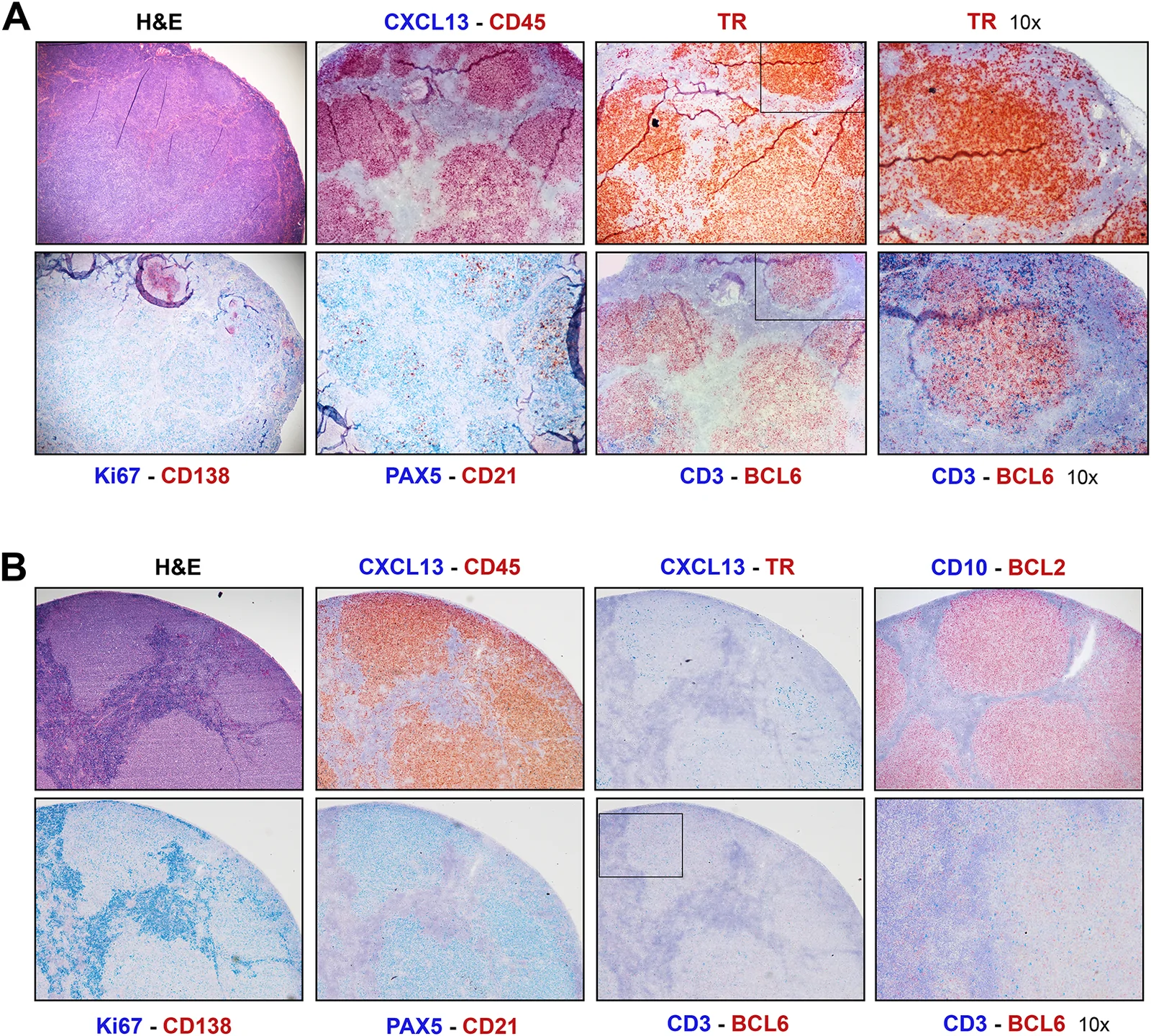
MHV68 induces virus-positive and virus-negative follicular B cell lymphomas. Representative images of **(A)** virus-positive and **(B)** virus-negative follicular B cell lymphomas. RNAscope in situ hybridization staining reveals follicular dendritic cell meshwork (CXCL13, CD21), leukocytes (CD45), B cells (PAX5), B cells positive for immunoglobulin light chain kappa (Igκ) or lambda (Igλ), plasma cells (CD138), T cells (CD3), germinal center B cells (BCL6), proliferation index (Ki67), BCL2-expressing cells, and virus-positive cells (TR). CD10 is not expressed in murine germinal center B cells. All images are shown at 4x magnification unless otherwise stated. Rectangles indicate the locations of 10x images.

Generally, MHV68-associated DLBCLs (**Figure 8A**) featured large atypical lymphoid cells with vesicular chromatin, prominent nucleoli, increased eosinophilic cytoplasm, and high proliferative activity, consistent with aggressive large B cell lymphoma. These tumors segregated into two groups with shared histopathological features. The first subgroup (**Figure 8A**, left side) exhibited nodular to coalescing nodular growth, strong BCL6 expression, and abundant TR-positive cells, with some tumors exhibiting partial plasmacytic or plasmablastic differentiation and possible residual follicular meshworks. This subgroup also demonstrated strong MEF2B staining, with variable IRF4 (**Supplementary Figure 3**). In contrast, the second subgroup (**Figure 8A**, right side) displayed predominantly diffuse sheets of large atypical cells with frequent mitotic figures, weak BCL6 expression, weak MEF2B expression alongside strong IRF4 expression (**Supplementary Figure 3**), and sparse or absent viral signal, consistent with a more post–germinal center–like aggressive B cell lymphoma phenotype.

**Figure 8:**
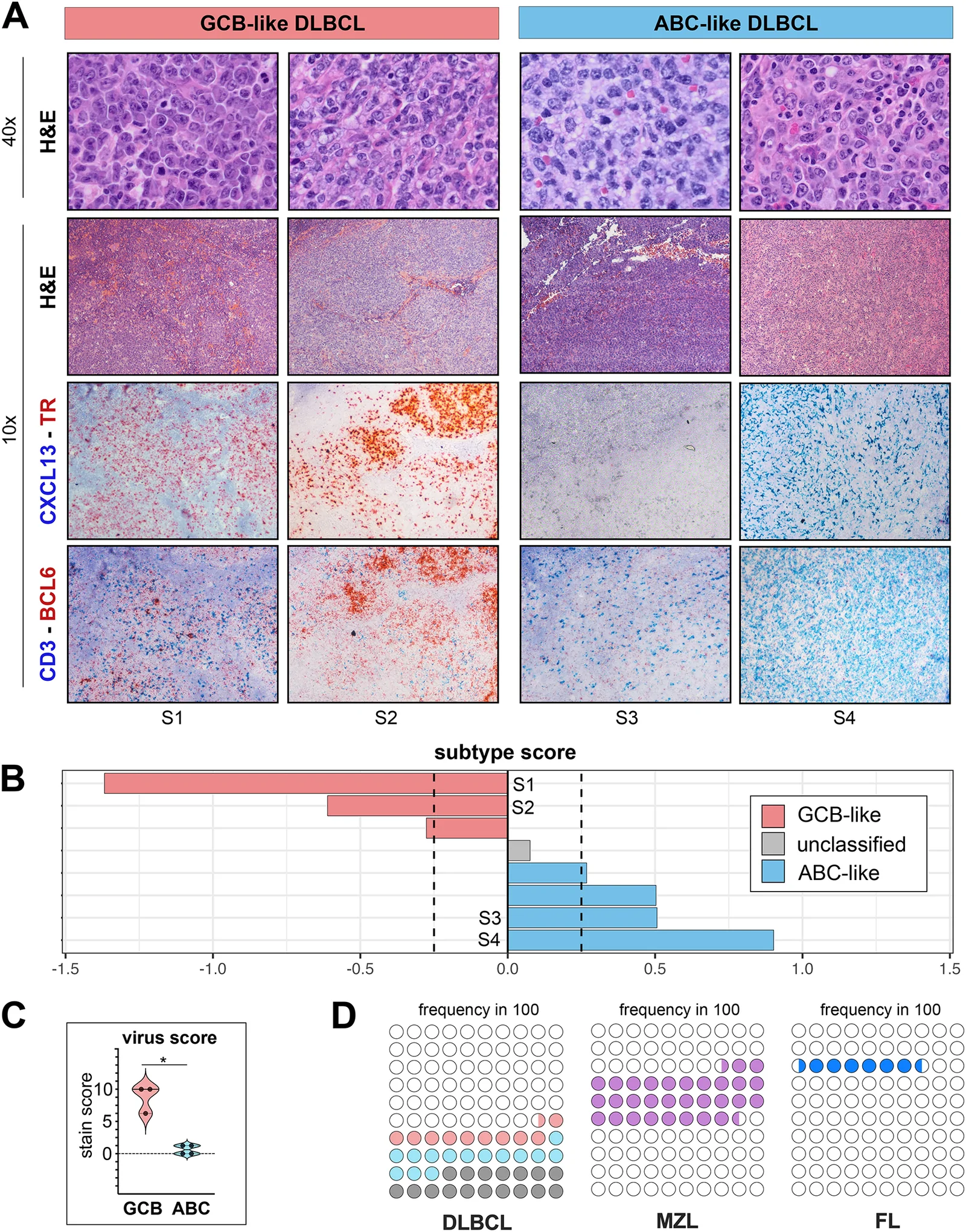
MHV68 induces diffuse large B cell lymphomas with histologic and transcriptional features of GCB-like and ABC-like DLBCL. **(A)** Representative images from four DLBCL. 40x magnification images (top row) show large atypical lymphoid cells. All other images are 10x magnification. RNAscope in situ hybridization staining reveals germinal center B cells (BCL6), T cells (CD3), and virus-positive cells (TR). CXCL13 staining was included only in the last panel of the CXCL13-TR row. Labels below the staining panels indicate sample numbers. GCB-like DLBCLs exhibited nodular architecture, strong BCL6 staining and abundant virus signal, whereas ABC-like DLBCLs displayed more diffuse morphology, weaker BCL6 staining, and low to absent virus signal. **(B)** Subtype scores of MHV68 DLBCLs based on the relative expression of genes associated with human ABC vs. GCB DLBCLs (adapted from classifier framework from Reddy et al. (24)). Sample numbers corresponding to the histology examples shown in panel **(A)** are indicated. **(C)** Quantification of virus scores of GCB-like vs. ABC-like DLBCLs. Each dot represents an individual mouse. Significance determined by two-tailed, unpaired Welch's t test (*p < 0.05). **(D)** Waffle plots depicting relative frequencies of DLBCL (GCB, ABC, unclassified), MZL, and FL among MHV68-associated B cell lymphomas. Each 100 circle plot represents the full B cell tumor cohort. Frequencies are based on n= 29 total, 12 DLBCL, 9 MZL, 2 FL, and 6 unclassified (not shown).

In humans, DLBCL comprises at least two major molecular subtypes: germinal center B cell–like (GCB) and activated B cell–like (ABC), which reflect distinct stages of B cell differentiation (germinal center versus post–germinal center) and exhibit different transcriptional programs and clinical behaviors (29). Although genetic profiling has further refined DLBCL classification into additional molecular groups (30, 31), gene expression signatures distinguishing ABC and GCB tumors remain the standard for DLBCL cell of origin classification (20, 32). To determine whether these histologically distinct MHV68 tumors correspond to known molecular subtypes of DLBCL, gene expression–based subtype scores were calculated based on the relative expression of genes associated with ABC versus GCB DLBCLs (24). This analysis indicated that MHV68 DLBCL subgroups corresponded to ABC-like and GCB-like transcriptional profiles (**Figure 8B**). Notably, GCB-like tumors exhibited a significantly higher abundance of virus-infected cells than ABC-like tumors (**Figure 8C**).

Across all evaluated tumors, DLBCL constituted the largest category of MHV68-induced B cell lymphomas (**Figure 8D**), with a frequency of 41.4% (n=12). MZL comprised the second largest category (31.0%; n=9), with follicular lymphomas occurring more rarely (6.9%; n=2). The predominance of DLBCLs, together with the recurrent presence of increased large cells within MZL tumors, suggests that a subset of MHV68-associated large B cell lymphomas may arise through transformation of lower-grade MZL. As described in **Figure 3**, the abundance of virus-positive cells was variable. Notably though, the divergent distribution of virus staining was apparent across all tumor subtypes (**Figure 9**), with most DLBCL, and MZL, FL, and unclassified B cell lymphomas segregating into subgroups of virus-rich or virus-poor subgroups (eg, virus-poor subgroup: DLBCL ABC, n=4/12; MZL group B, n=4/9; FL group B, n=1/2; unc. group B, n=3/6).

**Figure 9:**
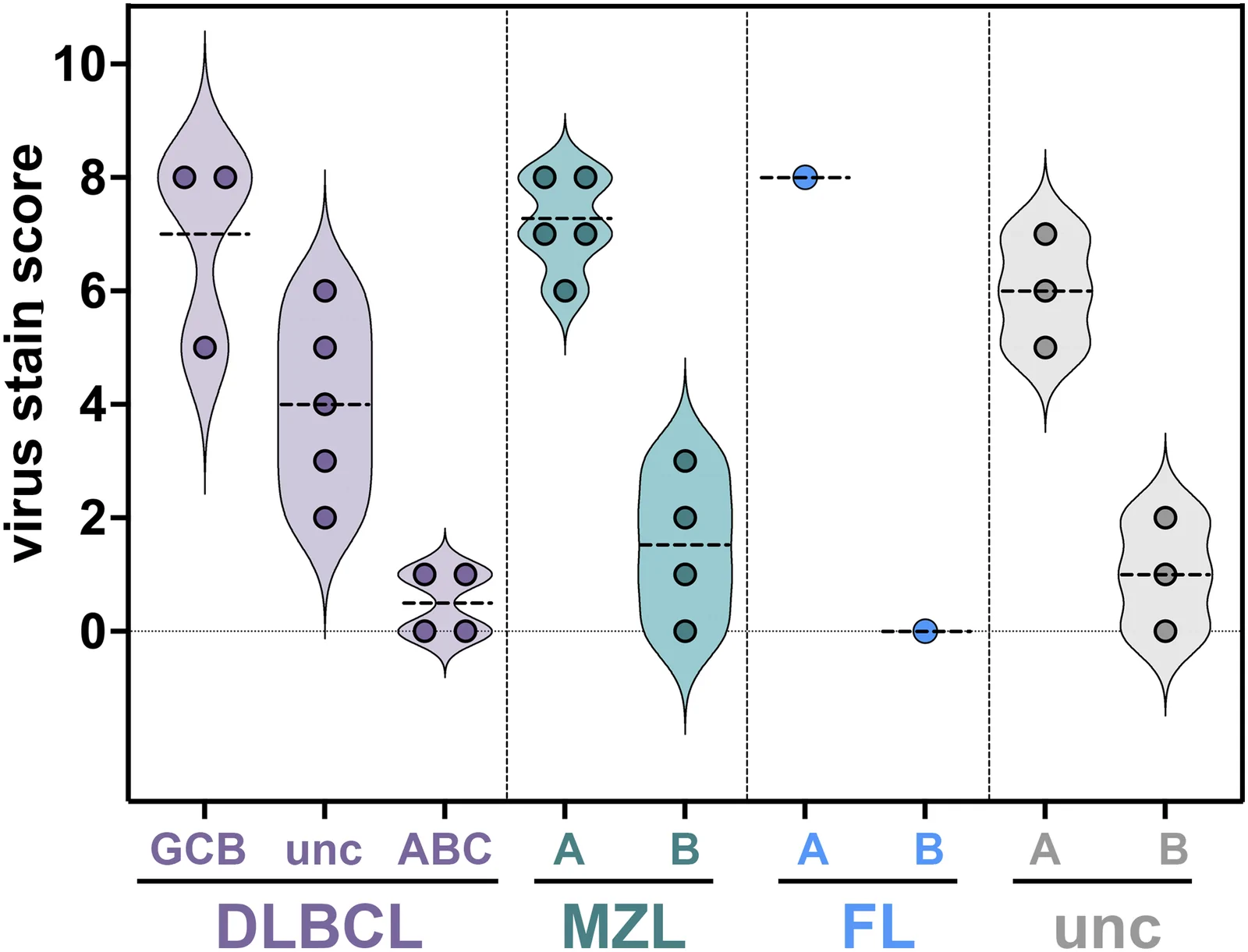
MHV68 B cell lymphomas segregate into virus-rich and virus-poor groups. Virus staining scores of subgroups of MHV68-associated DLBCL, MZL, FL, and unclassified (unc) B cell lymphomas. MZL (n= 9), FL (n= 2), and unclassified B cell lymphomas (n= 6) were segregated based on virus scores of 5-8 (A subgroups) and 0-2 (B subgroups). DLBCL (n= 12) were segregated based on GCB and ABC subtype scores as described in **Figure 8**, or were grouped as DLBCL unclassified (unc). Each dot represents an individual mouse. Dotted line represents the mean for each subgroup.

Together, these data demonstrate that MHV68 infection induces a heterogeneous spectrum of lymphoid malignancies. The majority are germinal center-derived B cell lymphomas that recapitulate morphological and immunophenotypic features of human EBV-associated DLBCL, MZL, and FL. Viral genome burden varies widely among tumors, and a subset exhibits evidence of monotypic expansion and widespread disease. This model therefore captures the histologic diversity and biological complexity characteristic of human gammaherpesvirus-associated lymphomas.

## Discussion

This study defines the major pathological features of MHV68-associated malignancies, and reveals that, even among genetically identical hosts, chronic gammaherpesvirus infection produces a diverse spectrum of B cell malignancies dominated by germinal center–derived B cell lymphomas. While previous work established that MHV68 infection can promote lymphoproliferative disease and lymphoma in immunodeficient settings (13, 14), the differentiation states and biological characteristics of the resulting tumors have remained incompletely defined. The findings presented here reveal that MHV68-associated malignancies encompass a heterogeneous spectrum of B cell neoplasia, including DLBCL, MZL and FL. The presence of immunoglobulin light chain restriction and systemic dissemination further demonstrates that these lesions represent clonal malignancies rather than reactive lymphoid proliferations. Together, these findings (a) support the concept that the germinal center is a critical mutational niche in which gammaherpesvirus persistence in B cells and malignant transformation intersect, and (b) highlight the essential role of immune surveillance in constraining deleterious outcomes from this niche.

A key biological feature emerging from this analysis is that the majority of B cell lymphomas exhibit a germinal center B cell phenotype, or derive from post-germinal center B cells (eg, ABC DLBCL) (29). Both EBV and MHV68 establish long-term latency within B cells that transit the germinal center reaction (6, 7, 10, 33, 34). Many EBV-associated B cell malignancies arise from germinal center (eg, Burkitt lymphoma, classic Hodgkin lymphoma) or post–germinal center B cells, particularly in settings of impaired immune surveillance (7, 29, 34). Thus, the predominance of germinal center–derived tumors observed following MHV68 infection is consistent with the cellular differentiation states observed in human gammaherpesvirus-associated lymphomas. These findings suggest that the virus-driven differentiation program that sustains gammaherpesvirus latency *in vivo* may also create conditions permissive for malignant transformation.

The germinal center reaction represents a highly specialized microenvironment characterized by intense proliferation, mutagenesis, and clonal selection. While these processes are essential for immunoglobulin affinity maturation, they also create conditions in which genetic errors can accumulate and promote malignant transformation (35). The frequent emergence of BCL6/MEF2B-positive malignancies in MHV68-associated disease raises the possibility that gammaherpesvirus perturbation of normal germinal center dynamics may directly contribute to lymphomagenesis. For example, viral manipulation of B cell biology may result in prolonged germinal center residence or delayed differentiation into memory or plasma cells (4, 6, 7, 36). Consistent with this concept, MHV68 encodes multiple factors that reshape the behavior of germinal center B cells. Viral proteins and noncoding RNAs modulate specific host pathways to promote GC B cell infection and latency: mLANA degrades NF-κB p65 and stabilizes MYC (37, 38), *mghv-miR-M1-7-5p* represses EWSR1 (39), the conserved viral protein kinase ORF36 cooperates with IRF-1 (40), and M2 functions in AID-expressing B cells and triggers p53 (41, 42). *In vivo* studies have demonstrated that the latent reservoir in GC cells is also supported by intrinsic B cell factors, including SHP1, Cul4b, STAT1 and STAT3 (43–46). In this scenario, gammaherpesvirus infection may effectively expand or prolong the mutational niche of the germinal center reaction, thereby increasing the probability that transforming host mutations arise during chronic infection.

An additional insight from this study is the striking heterogeneity in viral genome abundance observed across MHV68-associated lymphomas. While some tumors contained abundant virus-positive cells, others exhibited sparse or patchy virus staining. This heterogeneity was present across all tumor subtypes, with DLBCL, and MZL, FL, and unclassified B cell lymphomas all segregating into virus-rich or virus-poor subgroups, suggesting that this observation may reflect distinct stages of tumor evolution rather than stochastic variability. This pattern parallels a long-recognized feature of EBV-associated malignancies in humans, in which viral genome abundance can vary widely between tumor types and even among cells within individual tumors (7, 34). Although the pattern observed for MHV68 DLBCL subtypes differs from that typically observed in clinical settings of human DLBCL in which EBV-positive cases are more frequently ABC/non-GCB category (3–5), this relationship is not absolute: in some human cohorts, as many as 40% of EBV-positive DLBCLs are classified as GCB type, and most ABC DLBCLs remain EBV-negative (3–5). The identification of both virus-rich and virus-poor MHV68-associated B cell lymphomas suggests that such divergence may represent a broader feature of the evolution of gammaherpesvirus-associated B cell tumors.

The observation here of both virus-rich and virus-poor MHV68-associated tumors, which is consistent with previous observations (14, 47), supports the possibility that gammaherpes- infection may promote lymphomagenesis through both virus-dependent and virus-initiated mechanisms. In both situations, the virus may promote prolonged residence of infected B cells within the germinal center state, increasing the opportunity for deleterious mutations. In the virus-dependent scenario, viral latency programs directly contribute to maintenance of malignant cells by continuing to promote survival, proliferation, and immune evasion (4, 48). In the virus-initiated scenario, direct involvement of the virus in initiation of the tumor facilitates the emergence of clonally expanding B cell populations during tumor evolution. Once a malignant clone emerges, continued evolution, including the accumulation of new driver mutations, results in a tumor that is progressively less dependent on the virus.

Similar mechanisms of hit-and-run oncogenesis have long been proposed for tumor viruses; however, direct evidence in human tumors remains limited and controversial (7, 34, 49, 50). Nevertheless, recent work has strengthened this concept by showing that highly sensitive detection methods can identify EBV traces in malignancies previously classified as EBV-negative, and that such tumors may retain epigenetic and mutational features resembling EBV-positive disease (51–53). Together, these findings support the possibility that, in at least a subset of cases, viral loss may occur as tumors continue to evolve after an earlier virus-driven oncogenic state. The MHV68 system may provide a tractable *in vivo* model for testing whether mechanisms resembling hit-and-run oncogenesis contribute to gammaherpesvirus-associated B cell transformation.

The work described here provides a framework for future studies to dissect how gammaherpesviruses shape the emergence and progression of a spectrum of B cell malignancies, particularly in the setting of impaired immune surveillance. Our findings reveal that, even in genetically identical hosts, gammaherpesvirus infection can give rise to diverse pathological outcomes, ranging from lymphoproliferative disease to multiple B cell lymphoma subtypes. More broadly, this model provides an experimental path to address a central unresolved question in gammaherpesvirus oncology: whether some tumors that appear virus-low or virus-negative at diagnosis may have arisen from earlier virus-driven stages of malignant evolution.

## Data Availability

The original contributions presented in the study are publicly available at NCBI, accession number PRJNA1539829.
