# Supplementary material for: Impaired immune surveillance exposes the germinal center as a niche for gammaherpesvirus-associated B cell lymphomagenesis
Source: Front Immunol. 2026 Oct 16;17:1859970. doi: 10.3389/fimmu.2026.1859970 (PMC13701162; doi:10.3389/fimmu.2026.1859970)
Supplement: Supplementary file 1 [file Table1.pdf]

Supplementary Table 1. DLBCL subclassification log2 normalized

	UNC (S7)	GCB (S3)	ABC (S3)	GCB (S1)	GCB (S2)	ABC (S6)
**Bmf**	9.34298332	9.45052766	10.1324624	9.65291541	10.6418742	10.9304589
**Sh3bp5**	11.7169232	12.3763847	12.4839964	10.4371088	12.4500693	11.2519652
**Blnk**	11.6331546	12.2609694	12.5990841	8.65702194	12.4840566	10.3396922
**Il16**	12.5575651	12.4482743	13.5513179	11.7361354	13.2538454	12.4288503
**Irf4**	11.2013424	12.5937168	12.0645455	9.56193483	11.9965057	10.5619255
**Pim1**	13.5889131	14.3848111	13.6875994	13.9574911	12.6659065	14.0588913
**Ccnd2**	14.1666968	13.7092709	14.8041342	12.0902517	13.8472379	13.6619588
**Entpd1**	12.2936473	11.2541039	12.971858	10.8752782	11.6807514	11.8566848
**Fut8**	11.3894345	11.4273202	11.4599929	10.765819	11.8852587	11.8242557
**Ptpn1**	14.3408296	13.5194031	15.1972675	12.5044565	13.6638767	13.7480985
**Etv6**	12.55823	12.0337708	13.222256	12.1132008	12.0717587	12.876708
**Lmo2**	13.4878434	13.13131	13.258569	13.7109596	12.2931381	12.5629773
**Nek6**	10.0457326	9.79442429	10.6712468	8.62426097	10.4384251	10.5088058
**Dennd3**	11.3794368	12.2476422	12.7599217	11.8077035	12.7280343	11.1486314
**Mme**	9.67594856	8.70296719	8.54884212	9.79846007	9.54423563	10.7032748
**Bcl6**	10.5769942	12.7098459	10.4477337	10.1748213	12.4167818	11.3427414
**Lrmp**	11.011134	11.2597321	12.1963622	12.3055417	12.0467808	11.3386195
**Mybl1**	7.98272649	10.6814317	7.74804139	7.76303392	12.5598439	8.43276967
**Itpkb**	12.7862748	12.5356429	12.8481669	12.6914337	12.7306846	11.9585706

ABC (S4)	ABC (S5)
9.98279124	9.23007289
12.2701343	11.7612173
12.8321268	11.582593
12.9335216	12.6069535
11.7994905	10.2953971
13.4221983	14.4037173
14.6440494	14.7573483
12.4586587	12.5010979
12.155319	11.1932041
14.8528376	14.7327765
12.8804204	12.6996532
12.353475	13.2384985
10.1671234	10.1980235
11.7419852	11.5335412
9.62539445	7.84836432
10.9156535	10.9435863
11.6693207	11.3471297
9.09569133	7.77950967
12.0879083	12.4046316
